# Implementation of evidence-based mental health practices in criminal justice settings: Implications from a Hybrid Type I cost-effectiveness trial

**DOI:** 10.1186/1748-5908-10-S1-A76

**Published:** 2015-08-20

**Authors:** Jennifer E Johnson, Shannon Wiltsey-Stirman, Robert Stout, Ted Miller, Caron Zlotnick

**Affiliations:** 1 Division of Public Health, College of Human Medicine, Michigan State University, Flint, MI, 48502, USA; 2 Department of Psychiatry and Human Behavior, Brown University, Providence, RI, 02906, USA; 3 Women's Health Sciences Division, National Center for PTSD, VA Boston Healthcare System, Boston, MA, 02130, USA; 4 Department of Psychiatry, Boston University, Boston, MA, 02215, USA; 5 Decision Sciences Institute, Pacific Institute for Research and Evaluation, Pawtucket, RI, 02860, USA; 6 Pacific Institute for Research and Evaluation, Calverton, MD, 20705, USA; 7 Women and Infants Hospital, Providence, RI, 02905, USA; 8 Butler Hospital, Providence, RI, 02906, USA

## Abstract

Justice-involved individuals are an underserved population with extremely high rates of mental health disorders. Very little mental health D/I research has been conducted in the justice system to date. This paper reports implications for implementation of evidence-based mental health practices in justice settings from results of a Hybrid Type I trial (R01 MH095230), framed within Taxman's Evidence-Based Criminal Justice Interagency Implementation Model (CJ-IIM). Implementation data from this trial include: (1) cost-effectiveness of providing an evidence-based psychosocial intervention (group interpersonal psychotherapy; IPT) for major depressive disorder (MDD) in state prisons at a dose typical of community (i.e., weekly), rather than prison (monthly) settings (RCT of 180 state prisoners with MDD in 8 state prisons in 2 states); (2) acceptability of the evidence-based practice to prison mental health providers and administrators (based on a survey of n = 47), (3) provider attitudes and competencies, and (4) barriers and facilitators of implementation in this trial (from qualitative analysis of study process notes). Preliminary effectiveness results indicated advantages for IPT for depressive symptoms and hopelessness, with some suggestion that effects were larger as providers gained experience with the intervention; cost-effectiveness analyses are ongoing. Survey results indicated that providers and administrators viewed MDD among prisoners as an important problem and perceived current strategies for treating MDD in prison as insufficient. They tended to be oriented toward rehabilitation rather than punishment and were friendly toward evidence-based practices. However, mental health budgets that were small relative to the magnitude of inmate treatment needs translated into heavy case loads and a focus on crisis management, with inconsistent supervision and staff and leadership turnover at some facilities, posing serious barriers to adoption and sustainability. Overall, prisoners are desperate for adequate care and their providers are friendly toward innovation and desperate for ways to provide prisoners with more adequate care. However, public investment in offender health is low and therefore resource and system barriers are substantial. We discuss the implications of these findings for system-wide implementation of evidence-based mental health practices, including public media messaging and cost-effectiveness research as potential ways to address systemic barriers.

